# Mind Reading

**Published:** 1891-02

**Authors:** 


### HALL'S JOURNAL OF HEALTH

TRUTH DEMANDS NO SACRIFICE; ERROR CAN MAKE NONE.

Vol. 38. FEBRUARY, 1891. No. 2.

### MIND READING.

It is too late in the day to dispute the existence of that wonderful faculty of the human brain, commonly called mind reading. There have been too many exhibitions of it, both public and private, under conditions which precluded the possibility of falsehood or deceit to leave its actuality an open question.

Writing upon this interesting subject the Editor of *London Light*, widely known under the *nom-de-plume* of M. A. (Oxon), says of it :

The gift of thought reading, so notable in the case of Irving Bishop, and, more recently in the case of Johnstone, is closely akin so that of mediumship. That which enabled Bishop and Johnstone to do what they did was, probably, a special psychical faculty stimulated to a very high degree. So stimulated, it was fatal in one case, and produced in the other similar symptoms which only just stopped short of death. There is no apparent reason to invoke the intervention of an alien spirit in these cases. The effects were probably due to an exaltation in an abnormal degree of the innate powers of the incarnate spirit in each case. With this in mind we may ask how much of the phenomena of mediumship may be referred to the action of the spirit of the medium, or of the circle, or of individuals composing it.

Again, it is abundantly clear that the excessive use of these mysterious powers is very dangerous to health, and even to life. The parallel holds here, too, in respect of the undue exercise of mediumship. Hudson Tuttle (an American mind reader, writing in the *Banner of Light*), tells us that he has found the exercise of his mediumship in unpleasant surroundings, *i. e.*, with those who do not understand its delicate conditions, or in the presence of a dominant antagonistic influence, so painful that he looks on it with dread. "When writing, sudden interruption is like a blow, it leaves me dazed and irritable." He says, moreover, that the excessive exercise of his mediumship is very deleterious. "Once * * * having written nearly all the night, till the pen had fallen from my fingers in the middle of a sentence, the result was disastrous. At nine in the morning I had a congestive chill * * * " from depleted vitality.

I am able to corroborate Mr. Tuttle's experience. In old days when our sittings at the late Dr. Stanhope Speer's were fruitful of such remarkable physical phenomena, I have been so depleted that the spinal column was no longer able to support the body. Sitting in my chair, unconscious, the upper part of the body gradually fell over to the right side, just as a candle placed too near the fire, would become limp and droop. I have risen from a seance, in which certain manifestations were more than ordinarily pronounced, feeling so weak as to be unable to walk alone. Even when automatic writing has been too much prolonged I have been so depleted of vitality that nothing but sleep, heavy and long, has relieved the desperate weight at the base of the brain and the weakness of the spine.

The fate of Irving Bishop, still fresh in the minds of our readers, affords a sad and startling corroboration of the conjoint testimony of these two above named writers and mind readers. It is high time that this subject should be intelligibly enquired into and its conditions understood and appreciated.

Nothing is gained by resisting obvious facts, by the cry of fraud. This is the last resort of bigots and ignoramuses.

